# Fatty Acid Profiles of Serum Lipid Fractions Change Minimally in Sled Dogs Before and After Short Bouts of Exercise

**DOI:** 10.3389/fvets.2021.704770

**Published:** 2021-08-23

**Authors:** James R. Templeman, Luciano Trevizan, David W. L. Ma, Anna K. Shoveller

**Affiliations:** ^1^Department of Animal Biosciences, University of Guelph, Guelph, ON, Canada; ^2^Department of Animal Science, Universidade Federal do Rio Grande do Sul, Porto Alegre, Brazil; ^3^Department of Human Health and Nutritional Sciences, University of Guelph, Guelph, ON, Canada

**Keywords:** endurance exercise, lipid metabolism, Siberian huskies, lipid fraction, lipid mobilization

## Abstract

Although emerging data suggests a greater influence of gluconeogenic precursors, endurance sled dogs have long appeared to rely heavily on fatty acid oxidation for sustained energy production. However, much of the research investigating lipid utilization during exercise in sled dogs has been carried out with dogs subjected to extended bouts of endurance exercise. Less is known about changes in fatty acid composition in endurance training sled dogs subjected to short bouts of exercise, and fewer data define how fatty acid composition may change in distinct lipid fractions. As such, the study objective was to assess whether short bouts of submaximal exercise would affect fatty acid profiles of serum lipid fractions in endurance training sled dogs. Fifteen privately-owned Siberian huskies were used (8 females: 4 intact, 4 spayed; 7 males: 2 intact, 5 neutered), with an average age of 4.6 ± 2.5 years and body weight of 24.8 ± 4.2 kg. Throughout the diet acclimation and remainder of the study, all dogs were fed a dry extruded diet that met or exceeded all AAFCO nutrient recommendations. Dogs were weighed weekly and fed to maintain baseline body weight. A 12-week exercise regimen was designed to incorporate weekly increases in running distance, but weather played a role in setting the daily distance. On weeks 2, 5, and 11, an exercise challenge was implemented whereby dogs would run 4 km at 15 km/h in teams of 4. Pre- and post-exercise blood samples were taken, and gas chromatography was used to evaluate fatty acid profiles of all identified serum lipid fractions (cholesterol ester, diacylglycerol, free fatty acid, phospholipids, triglyceride). Data were analyzed using PROC MIXED of SAS, with dog as a random effect and week and sampling time point as fixed effects. Composition of oleic (18:1n9), linoleic (18:2n6), and alpha-linolenic (18:3n3) acids in the free fatty acid fraction decreased by ~9, 10, and 60%, respectively, following exercise (*P* ≤ 0.05). The results presented herein suggest that aside from a degree of depletion of these 18-carbon unsaturated fatty acids, short bouts of submaximal exercise do not induce considerable changes to sled dog fatty acid profiles.

## Introduction

Largely due to the insulative properties of their coats decreasing thermoregulatory energy expenditure at low environmental temperatures, the maintenance energy requirements of Siberian huskies are similar to or even lower than many other breeds (1, 2). However, as level of exercise and energy expenditure are positively associated (3), the requirement for dietary energy will increase as exercise increases in duration and intensity (3). Due to the extended duration of the exercise performed, combined with the fact that sled dogs are typically housed, trained and raced outdoors during months where the ambient temperature is below the thermoneutral zone of these breeds (4, 5), racing sled dogs can require upwards of 1,000 kcal/kg of metabolic body weight per day (6). As such, adequate provision of dietary energy is a critical aspect of nutritional programs developed for sporting dogs undergoing endurance-style training and competition, especially at sub-thermoneutral temperatures.

Depending on the duration and intensity of physical exertion, exercising dogs will utilize several sources of energy to support the demands of working muscle (1). While contributions from the phosphagen system, anaerobic and/or aerobic glycolysis support the immediate energy demands of working muscle, lipid oxidation has been recognized as a principal source of metabolic fuel for endurance-style athletes (7, 8). Endurance racing sled dogs have long appeared to rely heavily on the oxidation of fatty acids (FA) for sustained energy production, and in fact, the contribution of lipid oxidation to total energy production of dogs is twice that of humans, both at rest and during exercise (9, 10). This is due, in part, to the greater affinity for albumin to bind free fatty acids (FFA) in dogs relative to less aerobic species such as humans or goats (11–13), since transport of exogenous FA into working muscle relies on albumin binding (14). Dogs also have a greater mitochondrial volume and capillary red cell volume than goats, which likely enhances the oxygen-transporting capacity of the exercising dog. Furthermore, in addition to possessing greater intracellular stores of both glycogen and lipids, lipid droplets appear to be more tightly associated with mitochondria in the dog than the goat (13). Moreover, while there are a variety of hormones responsible for the regulation of substrate metabolism during a bout of exercise, the catecholamines norepinephrine and epinephrine play a predominant role in initiating the FA oxidation cascade in the adipocyte (14). Serum concentrations of both norepinephrine and epinephrine increased above resting levels in dogs subjected to as little as 5 min of moderate intensity exercise, with the exercise-induced response of epinephrine appearing to be greater in dogs than in humans (15). This may also lend some indication as to greater capacity for dogs to mobilize and oxidize fats compared to less aerobic species like humans, at least during exercise.

Feeding sled dogs high fat diets can increase resting levels of serum triglycerides (TAG) and FFA to be used as oxidative fuel sources (16), which can “spare” the use of muscle glycogen, therefore delaying the onset of fatigue related with muscle glycogen depletion (17, 18). As such, sled dogs have traditionally been fed high fat diets; however, more recent research suggests that sled dogs may in fact rely on carbohydrate oxidation during extended submaximal exercise, sustained by gluconeogenic precursors (e.g., amino acids, glycerol, lactate), even when fed low-to-moderate carbohydrate diets (19, 20). Although sled dogs are experiencing FFA depletion when subjected to extreme distance (e.g., 1,500+ km) submaximal exercise (20, 21), based on serum metabolomic data from Alaskan huskies, it appears as though prolonged aerobic exercise may create a beta-oxidation bottle neck, as accumulation of carnitine-bound FA (binding required for mitochondrial translocation) was evident, particularly midway (~775 km) through a multi-day ultra-endurance race (20).

Much of the research that has helped shape our understanding of lipid utilization during exercise in sled dogs (16, 18–23) has been done under conditions of long-distance races or training bouts of extended duration; however, less is known regarding changes in FA profiles in endurance training sled dogs subjected to short bouts of exercise. Moreover, as incremental conditioning regimens, which are comprised of exercise duration and/or intensity increasing in a stepwise manner, have long been implemented for endurance training athletes (24–28), improving our understanding of lipid metabolism and utilization during short and extended bouts of submaximal exercise is imperative. Finally, although more labor intensive than determining total serum lipid, the assessment of FA in circulating lipid fractions is valuable as these pools will all differ in FA composition, and as such may reflect tissue level changes in response to metabolic perturbations (29). Examining the effects of interventions, such as exercise, on total serum lipids may mask changes occurring in any single lipid fraction, potentially leading to inaccurate conclusions (30). As such, the objective of this current study was to investigate whether short bouts of submaximal exercise (4 km at 15 km/h) would affect FA profiles of serum lipid fractions in endurance training sled dogs. We hypothesized that exercise bouts of this duration and intensity would not induce changes in FA composition of any serum lipid fraction.

## Materials and Methods

### Animals and Housing

The present experiment was approved by the University of Guelph's Animal Care Committee (animal use protocol #4008). Fifteen client-owned domestic Siberian huskies (8 females: 4 intact, 4 spayed; 7 males: 2 intact, 5 neutered), with an average age of 4.6 ± 2.5 years and body weight (BW) of 24.8 ± 4.2 kg, were used in the study. Sixteen dogs were enrolled in the study for exercise purposes (so as to balance the gangline); however, due to an aversion to the restraint and collection procedure, no blood samples were collected from one dog (spayed female) throughout the trial period. Dogs were housed and trained at an off-site facility (Rajenn Siberian Huskies, Ayr, ON) that had been visited and approved by the University of Guelph's Animal Care Services. During the study, dogs were pair or group-housed in free-run, outdoor kennels that ranged in size from 3.5 to 80 square meters and contained between 2 and 10 dogs each. Two dogs were removed from the trial (one neutered male on week 7, one neutered male on week 9) due to exercise-related injuries; all data collected from them up until their respective points of removal are included.

### Diets and Study Design

Throughout the diet acclimation period (4 weeks) (16, 31, 32) the remainder of the study period (10 weeks), all dogs were fed a dry extruded diet (Champion Petfoods LT., Morinville, AB) that met or exceeded all NRC (3) and AAFCO (33) nutrient recommendations (Table 1) and were fed once daily at 17:00 h. Diet intake levels at the beginning of the acclimation period were determined from historical feeding records; BW was recorded for each dog at the beginning of the acclimation period and measured each week subsequently. Food allotments were adjusted to maintain the dogs' initial BW. For detailed information regarding diet intake and BW throughout the study period, refer to Templeman et al. (27). At feeding, all dogs were tethered and fed individually to allow for monitoring/measurement of diet consumption. Any orts were weighed and recorded daily. Throughout the entire trial period, all dogs were allowed *ad libitum* access to fresh water.

**Table 1:** Diet nutrient content on dry matter basis and ingredient composition^1^.

**Nutrient contents**	**Analyzed content**
Metabolizable energy, kcal/kg (calculated)^2^	4074.35
DM^3^, %	94.15
CP, %	47.92
CF, %	2.43
Ash, %	9.08
EE, %	24.16
**Fatty acids^4^, %**	
12:0	0.01
14:0	0.32
16:0	5.39
18:0	1.61
16:1n7	1.33
18:1n9	9.57
20:1n9	0.22
22:1n9	0.22
18:2n6	3.69
18:3n6	0.04
20:2n6	0.05
21:2n6	0.05
20:3n6	0.02
20:4n6	0.20
22:2n6	<0.01
22:4n6	0.03
22:5n6	0.02
18:3n3	0.39
18:4n3	0.11
20:3n3	0.01
20:4n3	0.02
20:5n3	0.30
22:3n3	0.05
22:5n3	0.05
22:6n3	0.45

1 *Ingredient composition: Pea starch, pork meal, fresh chicken, low ash herring meal, chicken fat, chicken meal, chicken skin meal, fresh pork, fresh pork organ blend^5^, chicken and turkey giblets, spray dried pork liver, pork-based palatant (liquid), hydrolyzed poultry protein, herring oil, pork-based palatant (dry), sodium chloride, dried kelp, choline chloride, poultry-based palatant, alpha tocopherol, natural antioxidant (liquid), thiamine, pantothenic acid, potassium chloride, selenium, zinc proteinate, natural antioxidant (dry), copper proteinate*.

2 *Metabolizable energy based on modified Atwater values and presented on an as-fed basis*.

3 *DM, dry matter (presented on an as-fed basis); CP, crude protein; EE, ether extract; CF, crude fiber*.

4 *Fatty acids presented as percent of ether extract*.

*^5^Blend of 51–52% fresh pork meat and 48–49% fresh pork organs (liver, kidney, spleen)*.

### Exercise Regimen and Exercise Challenges

A 12-week exercise regimen (2 weeks during diet acclimation, 10 weeks thereafter) was proposed whereby exercise duration would increase incrementally. However, decisions regarding the distance ran each day and number of stops (e.g., for water) were made with consideration of the ambient temperature and humidity. Inclement weather also caused the removal of the exercise challenge proposed to take place on week 8. Training consisted of dogs running on a standard 16-dog gangline with the gangline attached to an all-terrain vehicle with one rider who controlled the machine in its lowest gear. A pace of 15 km/h was averaged throughout the training period. Running pace and distance traveled was measured using a digital speedometer and odometer on the all-terrain vehicle. On weeks 2, 5, and 11, one off-day (no training) was replaced by an exercise challenge whereby dogs would run a consistent distance at a consistent pace as a team of 4 dogs. Four-dog teams were predetermined based on each dogs' position in the 16-dog gangline. All challenge groups for all weeks (2, 5, and 11) ran for 4 km at 15 km/h, with exercise challenges commencing at 07:00 h. In brief, dogs ran ~80 km total in total during the first 2 weeks, and ~1,250 km total over the next 10 weeks. For additional details regarding the exercise regimen (e.g., anticipated and actual daily/weekly run distances) or exercise challenges (e.g., determination of challenge distance), refer to Templeman et al. (27).

### Blood Sample Collection and Analysis

On exercise challenge days (weeks 2, 5, and 11), 5 ml pre-challenge blood samples were taken *via* cephalic venipuncture with a serum Vacutainer^®^ system (Becton, Dickinson and Company, Franklin Lakes, NJ, USA) following an overnight fast. Pre-exercise samples were taken 15 min prior to performing the exercise challenge (06:45 h). Once the challenge commenced (~16 min in duration), dogs were immediately watered and then had a post-exercise blood sample taken (~07:20 h) using the same procedure as stated above. All blood samples were centrifuged at 2,000 × g for 20 min at 4°C using a Beckman J6-MI centrifuge (Beckman Coulter, Indianapolis, IN), then serum aliquots were collected, frozen and stored at −80°C prior to analysis. Serum samples were analyzed for FA content and FA fractions using gas chromatography as described below.

Lipid extraction was done using the Folch method (34) Serum samples were thawed on ice then 100 μl of serum was added to 900 μl 0.1 M KCL (Sigma-Aldrich, Oakville, ON). A freshly prepared 2:1 CHCl_3_: MeOH (Sigma-Aldrich, Oakville, ON) mixture was added and the samples were vortexed, flushed with nitrogen, and kept overnight at 4°C. Samples were centrifuged at 357 × g for 10 min at 21°C using a Beckman J6-MI centrifuge (Beckman Coulter, Indianapolis, IN) and the chloroform layer was collected, dried down under nitrogen, and reconstituted with 100 μl of chloroform.

All samples were analyzed by lipid-class thin-layer chromatography (TLC). In brief, G-plates (EMD Chemicals Inc., Billerica, MS) were activated in an oven for 1 h at 100°C prior to being spotted by samples. Once spotted with the reconstituted samples, the G-plates were then placed in a TLC tank with freshly made TLC solvent containing 80 ml petroleum ether, 20 ml ethyl ether, and 1 ml acetic (Sigma-Aldrich, Oakville, ON). Following this, the plates were then lightly sprayed with 0.1% (w/v) 8-Anilino-1-naphthalenesulfonic acid ammonium (Sigma-Aldrich, Oakville, ON) before being visualized under UV light. Lipid class bands corresponding to cholesterol ester (CE), diacylglycerol (DAG), FFA, phospholipids (PL), and TAG were confirmed using authentic standards and collected for further analysis.

Methylation was performed by adding 2 ml of hexane and 2 ml of 14% BF_3_-MeOH (Sigma-Aldrich, Oakville, ON) to the samples and incubating them at 100°C for 60 min. Following methylation, 2 ml of double distilled H_2_O was added to the samples and the solution was immediately vortexed for 30 s to halt methylation. Samples were centrifuged at 357 × g for 10 min at 21°C using a Beckman J6-MI centrifuge (Beckman Coulter, Indianapolis, IN), the hexane layer was collected and dried down under nitrogen before reconstitution in 50 μl of hexane. Fatty acid methyl esters were quantified on an Agilent 6890 gas chromatograph (Agilent Technologies, Mississauga, ON) equipped with flame ionization detection and separated on an DB-FFAP fused-silica capillary column (15 m, 0.1 μm film thickness, 0.1 mm i.d.; Agilent Technologies, Mississauga, ON). Samples were injected in 200:1 split mode. The injector and detector ports were set at 250°C. Fatty acid methyl esters were eluted using a temperature program set initially at 150°C and held for 15 s, increased at 35°C/min and held at 170°C for 3 min, increased at 9°C/min to 225°C, and finally increased 80°C/min to 245°C and held for 2.2 min. The run time per sample is 12 min. The carrier gas was hydrogen, set to a 30 ml/min constant flow rate. Peaks were identified by retention times of FA methyl ester standards (Nu-Chek-Prep, Elysian, MN) using EZchrom Elite v. 3.2.1 software (Agilent Tech., Mississauga, ON). Fatty acid composition was calculated as percent (%) of total peak area.

### Statistical Analysis

Statistical analyses were performed with Statistical Analysis System (SAS) (v. 9.4; SAS Institute Inc., Cary, NC). All data were analyzed using PROC MIXED, where dog was treated as a random effect and week and sampling time point (pre- or post-exercise) were treated as fixed effects. Week was also treated as a repeated measure. When the fixed effects were significant, means were separated using the Tukey HSD. A Shapiro-Wilk test was used to assess normality and data were not transformed (*P* > 0.05). Statistical significance was declared at a *P* ≤ 0.05. Results are reported as least square means (LSM) ± standard error (SE) of the mean.

## Results

For the isolated CE fraction, no differences were identified between the pre- and post-exercise % composition for any FA for any week (Supplementary Table 1) or when pooled across weeks (*P* > 0.05, Table 2). With regard to differences observed with time, % composition of myristic (14:0; SFA), myristoleic (14:1; MUFA), palmitic (16:0; SFA), palmitoleic (16:1n9; MUFA), vaccenic (18:1n11; MUFA), linoleic (18:2n6; PUFA), and arachidonic (20:4n6; PUFA) acids all differed with week (*P* ≤ 0.05, Table 2). For each lipid fraction, only FA with mean % compositions of >5 will be discussed further; however, data for all FA identified for each fraction are described in Table 2 (CE), Table 3 (DAG), Table 4 (FFA), Table 5 (PL), and Table 6 (TAG). Percent composition of palmitic (16:0; SFA) acid was greater at week 11 than at weeks 2 and 5 (*P* ≤ 0.05, Table 2), but did not differ between weeks 2 and 5 (*P* > 0.05, Table 2). Linoleic (18:2n6; PUFA) acid % composition was greater at weeks 2 and 5 compared to week 11 (*P* ≤ 0.05, Table 2), but did not differ between weeks 2 and 5 (*P* > 0.05, Table 2). Arachidonic acid (20:4n6; PUFA) % composition was greater at week 2 than at weeks 5 and 11 and were greater at week 5 than week 11 (*P* ≤ 0.05, Table 2).

**Table 2:** Mean fatty acid composition (±SE^1^) and percent contribution of saturated fatty acids (SFA), monounsaturated fatty acids (MUFA), and polyunsaturated fatty acids (PUFA) for the cholesterol ester (CE) fraction identified in serum of all dogs at weeks 2, 5, and 11 (pooled across pre- and post-exercise sampling time points) and at the pre- and post-exercise sampling time points (pooled across weeks 2, 5, and 11).

**Fatty acid^2^, %**	**Week**			**Time point (TP)**		***P*** **-value**	
	**2**	**5**	**11**	**Pre**	**Post**	**Week**	**TP**
12:0	2.19 ± 0.43	1.30 ± 0.43	1.14 ± 0.49	1.97 ± 0.41	1.13 ± 0.41	0.18	0.09
14:0	0.09 ± 0.03	0.05 ± 0.03	0.06 ± 0.04	0.09 ± 0.04	0.05 ± 0.04	0.82	0.51
15:0	0.04 ± 0.01	0.03 ± 0.01	0.03 ± 0.02	0.03 ± 0.02	0.01 ± 0.02	0.36	0.24
16:0	10.56^b^ ± 0.35	11.14^b^ ± 0.35	15.04^a^ ± 0.39	12.25 ± 0.33	12.24 ± 0.33	<0.01	0.99
18:0	4.36 ± 1.07	5.47 ± 1.07	5.78 ± 1.18	5.65 ± 0.88	5.64 ± 0.88	0.22	0.99
19:0	0.05 ± 0.01	0.03 ± 0.01	0.04 ± 0.02	0.04 ± 0.01	0.03 ± 0.01	0.20	0.45
22:0	0.17 ± 0.12	0.19 ± 0.12	0.35 ± 0.14	0.27 ± 0.11	0.14 ± 0.11	0.11	0.21
23:0	0.14 ± 0.05	0.08 ± 0.05	0.03 ± 0.07	0.15 ± 0.05	0.07 ± 0.05	0.33	0.25
24:0	0.21 ± 0.10	0.24 ± 0.10	0.27 ± 0.13	0.24 ± 0.09	0.27 ± 0.09	0.60	0.88
14:1	0.41^a^ ± 0.07	0.12^ab^ ± 0.07	0.04^b^ ± 0.08	0.23 ± 0.06	0.15 ± 0.06	0.01	0.39
16:1n7	1.45^b^ ± 0.20	2.26^ab^ ± 0.20	2.76^a^ ± 0.25	2.02 ± 0.19	2.29 ± 0.19	<0.01	0.37
17:1n7	0.04 ± 0.01	0.03 ± 0.01	0.04 ± 0.02	0.04 ± 0.01	0.06 ± 0.01	0.46	0.35
18:1n7	3.35^a^ ± 0.10	3.06^ab^ ± 0.10	2.72^b^ ± 0.12	3.05 ± 0.10	3.03 ± 0.10	0.02	0.89
18:1n9	14.04 ± 0.35	12.67 ± 0.35	13.78 ± 0.41	13.45 ± 0.32	13.54 ± 0.32	0.42	0.53
20:1n11	0.10 ± 0.03	0.09 ± 0.03	0.12 ± 0.04	0.11 ± 0.03	0.09 ± 0.03	0.22	0.73
22:1n9	1.07 ± 0.21	0.92 0.21	1.15 ± 0.24	1.12 ± 0.20	0.98 ± 0.20	0.68	0.73
24:1	0.89 ± 0.12	0.68 ± 0.12	0.60 ± 0.13	0.68 ± 0.11	0.72 ± 0.11	0.40	0.71
18:2n6	50.86^a^ ± 1.43	49.57^a^ ± 1.43	42.32^b^ ± 1.61	43.87 ± 1.20	44.64 ± 1.20	<0.01	0.72
18:3n6	0.14 ± 0.05	0.16 ± 0.05	0.07 ± 0.06	0.11 ± 0.05	0.13 ± 0.05	0.47	0.23
20:2n6	0.04 ± 0.03	0.05 ± 0.03	0.10 ± 0.04	0.05 ± 0.03	0.08 ± 0.03	0.41	0.52
20:4n6	14.54^a^ ± 0.51	11.75^b^ ± 0.51	9.85^c^ ± 0.57	11.82 ± 0.45	12.26 ± 0.45	<0.01	0.48
22:4n6	0.03 ± 0.01	0.05 ± 0.01	0.15 ± 0.02	0.05 ± 0.01	0.08 ± 0.01	0.29	0.64
18:3n3	0.04 ± 0.05	0.14 ± 0.05	0.18 ± 0.06	0.14 ± 0.04	0.10 ± 0.04	0.15	0.51
20:5n3	1.52 ± 0.16	1.55 ± 0.16	1.81 ± 0.19	1.59 ± 0.15	1.42 ± 0.15	0.41	0.69
22:6n3	0.36 ± 0.15	0.41 ± 0.15	0.50 ± 0.17	0.42 ± 0.12	0.39 ± 0.12	0.64	0.66
Total SFA^3^	16.88 ± 0.89	18.13 ± 0.89	19.74 ± 1.01	20.16 ± 0.68	19.58 ± 0.68	0.58	0.71
Total MUFA	20.15 ± 1.00	19.88 ± 1.00	22.41 ± 1.13	18.12 ± 0.71	19.44 ± 0.71	0.55	0.68
Total PUFA	66.13^a^ ± 1.55	63.68^a^ ± 1.55	55.97^b^ ± 1.79	59.15 ± 1.41	59.81 ± 1.41	<0.01	0.82

1 *SE, standard error of the mean; n = 15 for weeks 2 and 5; n = 13 for week 11*.

2 *Any fatty acid(s) not detected within the specific fraction were removed from the table*.

3 *SFA, saturated fatty acids; MUFA, monounsaturated fatty acids; PUFA, polyunsaturated fatty acids*.

a, b, c *Values in a row (by week only) with different superscript are different (P ≤ 0.05)*.

**Table 3:** Mean fatty acid composition (±SE^1^) and percent contribution of saturated fatty acids (SFA), monounsaturated fatty acids (MUFA), and polyunsaturated fatty acids (PUFA) for the diacylglycerol (DAG) fraction identified in serum of all dogs at weeks 2, 5, and 11 (pooled across pre- and post-exercise sampling time points) and at the pre- and post-exercise sampling time points (pooled across weeks 2, 5, and 11).

**Fatty acid^2^, %**	**Week**			**Time point (TP)**		***P*** **-value**	
	**2**	**5**	**11**	**Pre**	**Post**	**Week**	**TP**
14:0	0.03 ± 0.02	0.03 ± 0.02	0.03 ± 0.03	0.05 ± 0.02	0.01 ± 0.02	0.98	0.11
16:0	28.13 ± 2.75	31.99 ± 2.75	31.77 ± 3.02	31.18 ± 2.26	30.08 ± 2.26	0.65	0.77
18:0	24.62 ± 2.41	22.2 ± 2.41	22.00 ± 2.68	23.22 ± 2.04	25.66 ± 2.04	0.76	0.11
19:0	0.22 ± 0.10	0.04 ± 0.10	0.07 ± 0.12	0.19 ± 0.08	0.21 ± 0.08	0.32	0.30
22:0	0.20 ± 0.16	0.27 ± 0.16	0.23 ± 0.18	0.25 ± 0.13	0.21 ± 0.13	0.51	0.60
23:0	0.22 ± 0.13	0.13 ± 0.13	0.03 ± 0.14	0.23 ± 0.12	0.16 ± 0.12	0.64	0.49
16:1n7	1.26 ± 0.84	1.77 ± 0.84	0.76 ± 0.93	1.85 ± 0.77	1.34 ± 0.77	0.88	0.56
18:1n9	18.12 ± 2.44	20.94 ± 2.44	20.83 ± 2.63	16.69 ± 2.04	20.57 ± 2.04	0.12	0.20
18:1n7	1.67 ± 0.87	1.05 ± 0.87	0.99 ± 0.92	0.66 ± 0.82	1.95 ± 0.82	0.24	0.18
20:1n11	0.02 ± 0.03	0.04 ± 0.03	0.03 ± 0.03	0.04 ± 0.03	0.02 ± 0.03	0.51	0.48
22:1n9	1.21 ± 0.55	1.35 ± 0.55	1.33 ± 0.73	1.54 ± 0.32	1.25 ± 0.32	0.68	0.53
24:1	1.04 ± 0.61	0.72 ± 0.61	0.66 ± 0.64	0.88 ± 0.54	0.73 ± 0.54	0.59	0.85
18:2n6	5.41 ± 1.07	5.49 ± 1.07	7.59 ± 1.22	6.06 ± 0.96	5.93 ± 0.96	0.06	0.40
18:3n6	0.21 ± 0.10	0.17 ± 0.10	0.06 ± 0.11	0.11 ± 0.09	0.24 ± 0.09	0.33	0.29
20:4n6	2.62 ± 0.66	1.78 ± 0.66	2.20 ± 0.74	2.75 ± 0.58	1.99 ± 0.58	0.83	0.06
18:3n3	0.01 ± 0.02	0.01 ± 0.02	0.02 ± 0.03	0.03 ± 0.02	0.01 ± 0.02	0.37	0.32
20:5n3	0.42 ± 0.16	0.35 ± 0.16	0.45 ± 0.17	0.41 ± 0.15	0.28 ± 0.15	0.66	0.52
22:6n3	0.39 ± 0.23	0.24 ± 0.23	0.28 ± 0.25	0.42 ± 0.20	0.24 ± 0.20	0.61	0.28
Total SFA^3^	57.82 ± 3.82	58.16 ± 3.82	55.73 ± 4.12	56.10 ± 3.13	56.33 ± 3.13	0.58	0.71
Total MUFA	24.44 ± 2.51	24.87 ± 2.51	25.60 ± 2.88	24.89 ± 1.96	25.86 ± 1.96	0.63	0.66
Total PUFA	10.66 ± 1.63	11.04 ± 1.63	11.16 ± 1.82	11.88 ± 1.44	9.96 ± 1.44	0.51	0.39

1 *SE, standard error of the mean; n = 15 for weeks 2 and 5; n = 13 for week 11*.

2 *Any fatty acid(s) not detected within the specific fraction were removed from the table*.

3 *SFA, saturated fatty acids; MUFA, monounsaturated fatty acids; PUFA, polyunsaturated fatty acids*.

**Table 4:** Mean fatty acid composition (±SE^1^) and percent contribution of saturated fatty acids (SFA), monounsaturated fatty acids (MUFA), and polyunsaturated fatty acids (PUFA) for the free fatty acid (FFA) fraction identified in serum of all dogs at weeks 2, 5, and 11 (pooled across pre- and post-exercise sampling time points) and at the pre- and post-exercise sampling time points (pooled across weeks 2, 5, and 11).

**Fatty acid^2^, %**	**Week**			**Time point (TP)**		***P*** **-value**	
	**2**	**5**	**11**	**Pre**	**Post**	**Week**	**TP**
14:0	0.20 ± 0.06	0.10 ± 0.06	0.10 ± 0.07	0.08 ± 0.05	0.18 ± 0.05	0.38	0.26
15:0	0.01 ± 0.01	0.02 ± 0.01	0.01 ± 0.02	0.01 ± 0.01	0.01 ± 0.01	0.45	0.39
16:0	23.61^b^ ± 0.64	25.03^b^ ± 0.64	30.03^a^ ± 0.72	27.62 ± 0.57	25.29 ± 0.57	<0.01	0.08
18:0	10.30^b^ ± 0.57	11.46^b^ ± 0.57	14.89^a^ ± 0.62	12.82 ± 0.50	11.65 ± 0.50	<0.01	0.06
24:0	0.05 ± 0.04	0.05 ± 0.04	0.04 ± 0.05	0.03 ± 0.04	0.07 ± 0.04	0.55	0.45
14:1	0.20 ± 0.07	0.18 ± 0.07	0.05 ± 0.08	0.13 ± 0.06	0.15 ± 0.06	0.31	0.79
16:1n7	3.58^c^ ± 0.31	4.64^b^ ± 0.31	5.69^a^ ± 0.34	4.34 ± 0.27	4.93 ± 0.27	<0.01	0.11
18:1n9	32.46 ± 0.86	29.31 ± 0.86	31.19 ± 0.99	32.66^†^ ± 0.77	29.30 ± 0.77	0.07	<0.01
18:1n7	3.55^a^ ± 0.18	2.64^b^ ± 0.18	2.57^b^ ± 0.20	2.84 ± 0.16	3.00 ± 0.16	<0.01	0.47
20:1n11	0.04 ± 0.03	0.03 ± 0.03	0.04 ± 0.03	0.02 ± 0.03	0.05 ± 0.03	0.33	0.27
22:1n9	0.76 ± 0.27	0.89 ± 0.27	1.10 ± 0.30	1.05 ± 0.25	0.73 ± 0.25	0.62	0.29
24:1	0.10 ± 0.07	0.06 ± 0.07	0.07 ± 0.08	0.12 ± 0.07	0.04 ± 0.07	0.25	0.28
18:2n6	17.61^a^ ± 0.44	13.17^b^ ± 0.44	12.14^b^ ± 0.47	15.04^†^ ± 0.38	13.57 ± 0.38	<0.01	<0.01
20:4n6	1.37 ± 0.22	1.22 ± 0.22	1.28 ± 0.24	1.44 ± 0.20	1.14 ± 0.20	0.86	0.18
22:5n6	2.37 ± 0.94	2.60 ± 0.94	4.34 ± 1.03	3.70 ± 0.81	2.59 ± 0.81	0.32	0.20
18:3n3	0.68^a^ ± 0.09	0.47^b^ ± 0.09	0.21^c^ ± 0.10	0.65^†^ ± 0.08	0.25 ± 0.08	<0.01	<0.01
20:5n3	0.06 ± 0.06	0.07 ± 0.06	0.04 ± 0.07	0.04 ± 0.05	0.07 ± 0.05	0.86	0.55
22:6n3	0.35 ± 0.11	0.32 ± 0.11	0.15 ± 0.12	0.23 ± 0.11	0.32 ± 0.11	0.23	0.34
Total SFA^3^	34.87^b^ ± 1.88	37.46^b^ ± 1.88	44.71^a^ ± 2.01	40.16 ± 1.46	39.01 ± 1.46	<0.01	0.40
Total MUFA	41.12 ± 1.51	38.25 ± 1.51	39.78 ± 1.66	39.86 ± 1.22	38.14 ± 1.22	0.28	0.33
Total PUFA	22.74 ± 1.27	18.85 ± 1.27	18.11 ± 1.54	20.52^†^ ± 0.66	18.77 ± 0.66	0.09	0.03

1 *SE, standard error of the mean; n = 15 for weeks 2 and 5; n = 13 for week 11*.

2 *Any fatty acid(s) not detected within the specific fraction were removed from the table*.

3 *SFA, saturated fatty acids; MUFA, monounsaturated fatty acids; PUFA, polyunsaturated fatty acids*.

a, b, c *Values in a row (by week only) with different superscript are different (P ≤ 0.05)*.

† *Mean value for the pre-exercise sampling point significantly differs from the post-exercise sampling time point within the same row (P ≤ 0.05)*.

**Table 5:** Mean fatty acid composition (±SE^1^) and percent contribution of saturated fatty acids (SFA), monounsaturated fatty acids (MUFA), and polyunsaturated fatty acids (PUFA) for the phospholipid (PL) fraction identified in serum of all dogs at weeks 2, 5, and 11 (pooled across pre- and post-exercise sampling time points) and at the pre- and post-exercise sampling time points (pooled across weeks 2, 5, and 11).

**Fatty acid^2^, %**	**Week**			**Time point (TP)**		***P*** **-value**	
	**2**	**5**	**11**	**Pre**	**Post**	**Week**	**TP**
14:0	0.09 ± 0.01	0.08 ± 0.01	0.08 ± 0.02	0.10 ± 0.02	0.07 ± 0.02	0.86	0.27
15:0	0.06 ± 0.01	0.07 ± 0.01	0.03 ± 0.01	0.06 ± 0.01	0.06 ± 0.01	0.14	0.63
16:0	15.66 ± 0.35	17.22 ± 0.35	16.40 ± 0.38	16.15 ± 0.30	16.70 ± 0.30	0.11	0.19
18:0	24.03^a^ ± 1.59	20.98^a^ ± 1.59	15.54^b^ ± 1.75	20.55 ± 1.39	17.81 ± 1.39	<0.01	0.15
19:0	0.15 ± 0.03	0.12 ± 0.03	0.07 ± 0.04	0.14 ± 0.02	0.12 ± 0.02	0.41	0.31
20:0	0.10 ± 0.02	0.11 ± 0.02	0.16 ± 0.03	0.12 ± 0.01	0.13 ± 0.01	0.21	0.23
22:0	0.08 ± 0.03	0.10 ± 0.03	0.17 ± 0.04	0.13 ± 0.02	0.15 ± 0.02	0.29	0.32
23:0	0.11 ± 0.02	0.12 ± 0.02	0.05 ± 0.03	0.09 ± 0.01	0.09 ± 0.01	0.31	0.79
24:0	0.12 ± 0.02	0.10 ± 0.02	0.13 ± 0.03	0.12 ± 0.02	0.14 ± 0.02	0.57	0.44
14:1	0.01 ± 0.01	0.02 ± 0.01	0.02 ± 0.01	0.02 ± 0.01	0.02 ± 0.01	0.91	0.99
16:1n7	0.53^b^ ± 0.15	0.66^b^ ± 0.15	1.27^a^ ± 0.17	0.87 ± 0.13	0.78 ± 0.13	<0.01	0.63
17:1n7	0.24 ± 0.03	0.29 ± 0.03	0.21 ± 0.04	0.25 ± 0.02	0.24 ± 0.02	0.61	0.85
18:1n7	3.04 ± 0.21	2.56 ± 0.21	2.55 ± 0.24	2.58 ± 0.18	2.85 ± 0.18	0.19	0.30
18:1n9	8.80^b^ ± 0.75	9.39^ab^ ± 0.75	10.52^a^ ± 0.82	9.14 ± 0.66	9.92 ± 0.66	0.01	0.36
20:1n11	0.21 ± 0.03	0.19 ± 0.03	0.18 ± 0.04	0.21 ± 0.03	0.20 ± 0.03	0.88	0.93
22:1n9	0.47 ± 0.12	0.49 ± 0.12	0.61 ± 0.13	0.54 ± 0.11	0.60 ± 0.11	0.51	0.74
24:1	1.02 ± 0.06	0.78 ± 0.06	0.61 ± 0.07	0.80 ± 0.05	0.84 ± 0.05	0.09	0.64
20:3n9	0.12^a^ ± 0.02	0.10^ab^ ± 0.02	0.06^b^ ± 0.02	0.10 ± 0.01	0.11 ± 0.01	0.01	0.28
18:2n6	26.32^a^ ± 1.26	24.47^ab^ ± 1.26	20.12^b^ ± 1.40	23.31 ± 1.05	23.24 ± 1.05	<0.01	0.97
18:3n6	0.15 ± 0.02	0.17 ± 0.02	0.10 ± 0.03	0.16 ± 0.02	0.12 ± 0.02	0.15	0.23
20:2n6	0.13 ± 0.02	0.16 ± 0.02	0.23 ± 0.03	0.17 ± 0.02	0.19 ± 0.02	0.14	0.29
20:3n6	1.03 ± 0.06	1.04 ± 0.06	0.70 ± 0.08	0.84 ± 0.05	0.92 ± 0.05	0.22	0.36
20:4n6	17.12^a^ ± 0.57	15.76^b^ ± 0.57	15.22^b^ ± 0.62	15.59 ± 0.49	16.35 ± 0.49	<0.01	0.28
22:4n6	0.20 ± 0.03	0.21 ± 0.03	0.28 ± 0.04	0.24 ± 0.02	0.26 ± 0.02	0.45	0.67
22:5n6	0.12 ± 0.01	0.10 ± 0.01	0.10 ± 0.01	0.10 ± 0.01	0.11 ± 0.01	0.26	0.30
18:3n3	0.30^a^ ± 0.03	0.31^a^ ± 0.03	0.17^b^ ± 0.04	0.29 ± 0.02	0.24 ± 0.02	<0.01	0.14
20:5n3	2.35 ± 0.15	2.66 ± 0.15	2.51 ± 0.17	2.51 ± 0.13	2.50 ± 0.13	0.49	0.97
22:5n3	0.61 ± 0.05	0.55 ± 0.05	0.75 ± 0.06	0.63 ± 0.04	0.71 ± 0.04	0.22	0.31
22:6n3	3.51 ± 0.24	2.35 ± 0.24	3.38 ± 0.27	2.99 ± 0.20	3.20 ± 0.20	0.11	0.21
Total SFA^3^	39.94^b^ ± 2.04	38.19^b^ ± 2.04	33.61^a^ ± 2.16	37.49 ± 1.89	35.57 ± 1.89	<0.01	0.26
Total MUFA	13.32 ± 1.18	14.33 ± 1.18	15.97 ± 1.38	13.92 ± 1.04	14.61 ± 1.04	0.14	0.22
Total PUFA	50.76^a^ ± 2.29	47.18^ab^ ± 2.29	45.71^b^ ± 2.40	48.55 ± 2.11	49.25 ± 2.11	<0.01	0.41

1 *SE, standard error of the mean; n = 15 for weeks 2 and 5; n = 13 for week 11*.

2 *Any fatty acid(s) not detected within the specific fraction were removed from the table*.

3 *SFA, saturated fatty acids; MUFA, monounsaturated fatty acids; PUFA, polyunsaturated fatty acids*.

a, b *Values in a row (by week only) with different superscript are different (P ≤ 0.05)*.

**Table 6:** Mean fatty acid composition (±SE^1^) and percent contribution of saturated fatty acids (SFA), monounsaturated fatty acids (MUFA), and polyunsaturated fatty acids (PUFA) for the triacylglycerol (TAG) fraction identified in serum of all dogs at weeks 2, 5, and 11 (pooled across pre- and post-exercise sampling time points) and at the pre- and post-exercise sampling time points (pooled across weeks 2, 5, and 11).

**Fatty acid^2^, %**	**Week**			**Time point (TP)**		***P*** **-value**	
	**2**	**5**	**11**	**Pre**	**Post**	**Week**	**TP**
14:0	0.43 ± 0.09	0.31 ± 0.09	0.20 ± 0.10	0.26 ± 0.08	0.37 ± 0.08	0.39	0.42
15:0	0.01 ± 0.02	0.02 ± 0.02	0.01 ± 0.02	0.01 ± 0.01	0.01 ± 0.01	0.30	0.57
16:0	20.71^b^ ± 1.02	21.51^b^ ± 1.02	30.75^a^ ± 1.19	24.87 ± 0.88	23.78 ± 0.88	<0.01	0.49
18:0	11.08^b^ ± 0.96	9.56^b^ ± 0.96	21.63^a^ ± 1.10	13.85 ± 0.82	14.33 ± 0.82	<0.01	0.76
20:0	0.13 ± 0.09	0.12 ± 0.09	0.25 ± 0.10	0.19 ± 0.08	0.16 ± 0.08	0.37	0.30
22:0	0.05 ± 0.05	0.04 ± 0.05	0.10 ± 0.06	0.09 ± 0.05	0.07 ± 0.05	0.45	0.66
24:0	0.16 ± 0.09	0.18 ± 0.09	0.07 ± 0.10	0.11 ± 0.08	0.17 ± 0.08	0.64	0.63
14:1	0.10 ± 0.10	0.18 ± 0.10	0.29 ± 0.11	0.24 ± 0.08	0.15 ± 0.08	0.41	0.44
16:1n7	2.40^b^ ± 0.47	3.63^ab^ ± 0.47	4.16^a^ ± 0.51	3.76 ± 0.42	3.57 ± 0.42	<0.01	0.71
18:1n7	3.55^a^ ± 0.30	3.75^a^ ± 0.30	2.48^b^ ± 0.33	2.74 ± 0.26	3.12 ± 0.26	<0.01	0.29
18:1n9	30.66^a^ ± 1.26	33.36^a^ ± 1.26	19.78^b^ ± 1.41	27.82 ± 1.09	26.06 ± 1.09	<0.01	0.29
20:1n11	0.04 ± 0.04	0.04 ± 0.04	0.05 ± 0.04	0.07 ± 0.03	0.03 ± 0.03	0.71	0.55
22:1n9	1.78 ± 0.96	3.67 ± 0.96	3.24 ± 1.07	2.87 ± 0.82	3.01 ± 0.82	0.41	0.87
24:1	0.16 ± 0.07	0.08 ± 0.07	0.07 ± 0.08	0.14 ± 0.07	0.05 ± 0.07	0.36	0.43
18:2n6	18.41^a^ ± 0.81	14.95^a^ ± 0.81	10.02^b^ ± 0.89	13.95 ± 0.68	13.64 ± 0.68	<0.01	0.77
20:3n6	0.15 ± 0.04	0.11 ± 0.04	0.13 ± 0.05	0.15 ± 0.04	0.10 ± 0.04	0.41	0.44
20:4n6	4.99 ± 0.59	3.37 ± 0.59	3.71 ± 0.63	3.80 ± 0.52	4.25 ± 0.52	0.10	0.48
22:4n6	0.01 ± 0.01	0.02 ± 0.01	0.01 ± 0.01	0.02 ± 0.01	0.01 ± 0.01	0.56	0.71
18:3n3	0.30 ± 0.07	0.24 ± 0.07	0.25 ± 0.08	0.28 ± 0.06	0.27 ± 0.06	0.28	0.66
20:5n3	1.95 ± 0.37	1.68 ± 0.37	1.82 ± 0.40	1.68 ± 0.32	1.95 ± 0.32	0.86	0.53
22:5n3	0.07 ± 0.05	0.07 ± 0.05	0.05 ± 0.06	0.08 ± 0.04	0.05 ± 0.04	0.96	0.63
22:6n3	2.29 ± 0.41	2.21 ± 0.41	1.94 ± 0.43	2.05 ± 0.35	2.25 ± 0.35	0.81	0.66
Total SFA^3^	32.67^b^ ± 2.32	31.71^b^ ± 2.32	53.01^a^ ± 2.40	39.58 ± 2.18	38.89 ± 2.18	<0.01	0.68
Total MUFA	38.74^a^ ± 2.65	43.19^a^ ± 2.65	29.07^b^ ± 2.88	37.13 ± 2.24	36.64 ± 2.24	<0.01	0.71
Total PUFA	28.57^a^ ± 1.80	23.96^b^ ± 1.80	18.94^b^ ± 1.97	22.97 ± 1.45	22.91 ± 1.45	<0.01	0.82

1 *SE, standard error of the mean; n = 15 for weeks 2 and 5; n = 13 for week 11*.

2 *Any fatty acid(s) not detected within the specific fraction were removed from the table*.

3 *SFA, saturated fatty acids; MUFA, monounsaturated fatty acids; PUFA, polyunsaturated fatty acids*.

a, b *Values in a row (by week only) with different superscript are different (P ≤ 0.05)*.

For the DAG fraction, no differences were identified between the pre- and post-exercise % compositions for any FA for any week (Supplementary Table 2) or when pooled across weeks (*P* > 0.05, Table 3). As well, no differences were observed with week for any FA (*P* > 0.05, Table 3).

For the FFA fraction, no differences were identified between the pre- and post-exercise % composition for any FA for any week (Supplementary Table 3); however, when these data were pooled, the pre-exercise % compositions of oleic (18:1n9; MUFA), linoleic (18:2n6; PUFA), and alpha-linolenic (18:3n3; PUFA) acids were greater than post-exercise (*P* ≤ 0.05, Table 4). For differences observed over time, % compositions of palmitic (16:0; SFA), palmitoleic (16:1n9; MUFA), stearic (18:0; SFA), vaccenic (18:1n11; MUFA), linoleic (18:2n6; PUFA), and alpha-linolenic (18:3n3; PUFA) acids all differed with week (*P* ≤ 0.05, Table 4). Percent compositions of palmitic (16:0; SFA) and stearic (18:0; SFA) acids were greater at week 11 than at weeks 2 and 5 (*P* ≤ 0.05, Table 4), but did not differ between weeks 2 and 5 (*P* > 0.05, Table 4). Percent composition of palmitoleic (16:1n9; MUFA) acid was greater at week 11 than at weeks 5 and 2 and was greater at week 5 than week 2 (*P* ≤ 0.05, Table 4). Linoleic (18:2n6; PUFA) acid % composition was greater at week 2 than at weeks 5 and 11 (*P* ≤ 0.05, Table 4), but did not differ between weeks 5 and 11 (*P* > 0.05, Table 4).

For the PL fraction, no differences were observed between pre- and post-exercise % composition for any FA for any week (Supplementary Table 4) or when pooled across weeks (*P* > 0.05, Table 5). With regard to differences observed with time, % compositions of palmitoleic (16:1n9; MUFA) and oleic (18:1n9; MUFA) increased with time. The % compositions of stearic (18:0; SFA), linoleic (18:2n6; PUFA), alpha-linolenic (18:3n3; PUFA), mead (20:3n9; PUFA), and arachidonic (20:4n6; PUFA) acids all decreased with week (*P* ≤ 0.05, Table 5). Percent composition of stearic (18:0; SFA) acid was greater at weeks 2 and 5 compared to week 11 (*P* ≤ 0.05, Table 5), but did not differ between weeks 2 and 5 (*P* > 0.05, Table 5). Oleic (18:1n9; MUFA) acid % composition was greater on week 11 than on week 2 (*P* ≤ 0.05, Table 5), but on week 5 did not differ from weeks 2 or 11 (*P* > 0.05, Table 5). Percent composition of linoleic acid (18:2n6; PUFA) was greater on week 2 compared to week 11 (*P* ≤ 0.05, Table 5), but on week 5 did not differ from weeks 2 or 11 (*P* > 0.05, Table 5). Arachidonic acid (20:4n6; PUFA) % composition as greater on week 2 than on weeks 5 and 11 (*P* ≤ 0.05, Table 5), but did not differ between weeks 5 and 11 (*P* > 0.05, Table 5).

For the TAG fraction, no differences were observed between pre- and post-exercise % compositions for any FA for any week (Supplementary Table 5) or when pooled across weeks (*P* > 0.05, Table 6). For differences observed with week, % compositions of palmitic (16:0; SFA), palmitoleic (16:1n9), stearic (18:0; SFA), oleic (18:1n9; MUFA), vaccenic (18:1n11; MUFA), and linoleic (18:2n6; PUFA) acids all differed with week (*P* ≤ 0.05, Table 6). Palmitic (16:0; SFA) and stearic (18:0; SFA) acid % compositions were greater at week 11 compared to weeks 2 and 5 (*P* ≤ 0.05, Table 6), but did not differ between weeks 2 and 5 (*P* > 0.05, Table 6). Oleic (18:1n9; MUFA) and linoleic (18:2n6; PUFA) acid % compositions were greater for weeks 2 and 5 than for week 11 (*P* ≤ 0.05, Table 6), but did not differ between weeks 2 and 5 (*P* > 0.05, Table 6).

## Discussion

Fatty acid profiles of serum lipid fractions of endurance training sled dogs were examined before and after short bouts of submaximal exercise during a multi-week endurance training regimen. The objective was to investigate whether these short exercise bouts affect sled dog FA profiles, as shifts in serum FA had been observed in sled dogs subjected to extended durations of submaximal exercise (16, 20, 21).

The results presented herein suggest that bouts of submaximal exercise of 15 min or less (4 km at 15 km/h) induce few changes to sled dog FA profiles. Composition of oleic (18:1n9; MUFA), linoleic (18:2n6; PUFA), and alpha-linolenic (18:3n3; PUFA) acids in the FFA fraction decreased by ~9, 10, and 60%, respectively, following exercise, suggesting a degree of depletion and/or tissue uptake of these 18-carbon unsaturated FA in response to these short runs. Gold et al. (35) subjected mongrel dogs to 20–25 min of submaximal exercise and reported reductions in serum concentrations of 18:1n9 and 18:2n6 FFA (~6 and 2%, respectively), but concentrations of 18:3n3 were not reported. These changes are less substantial than those observed in the current study, but the dogs used by Gold et al. (35) were running at approximately half the pace (6 km/h) than those in the current study. Unfortunately, little information was presented regarding the conditioning of the dogs prior to the exercise challenge, or the diets being fed (35). McClelland et al. (12) set out to investigate how or if the serum FFA responses to exercise would differ between two species of varying aerobic capacity: dogs and goats. The comparable aspect of this study was confounded by differences in dietary FA composition; however, comparison aside, when dogs were subjected to exercise demanding 40% of their maximal rate of oxygen consumption (VO_2_max), oleic acid (18:1n9) concentrations decreased by ~10% in serum by ~20–30 min of exercise. This decrease was short lived, though, as serum concentrations were greater than pre-exercise levels by ~100 min after exercise and staying elevated for up to 190 min post-exercise (12). Concentrations of linoleic acid (18:2n6) were unchanged after ~20–30 min of exercise, but the serum concentrations decreased by ~12% after 30 min and remained lower than pre-exercise levels during post-exercise recovery (12). Unfortunately, concentrations of 18:3n3 were also not reported and the breed of dog used was not disclosed by (12). As a note, the dogs in the current study were running at a pace of 15 km/h, which would be equivalent in relative intensity to ~50% of VO_2_max pace for moderately trained sled dogs (29.5 km/h) (36). Ultimately, these data suggest that changes to Siberian husky FA profiles induced in response to short bouts of submaximal exercise are similar in specific FFA and in magnitude of change to that of other breeds that would be used less prominently for endurance sled dog racing, but may be used for different types of endurance-style activity, such as hunting, search and rescue or scent detection (37, 38).

Although short bouts of exercise only minimally affected FA profiles, changes were observed as dogs progressed through training in the present study. Noteworthy outcomes included decreasing serum % composition of linoleic (18:2n6) and arachidonic (20:4n6) acids. Linoleic acid, an essential FA for all mammals including dogs (39), is a metabolic precursor of arachidonic acid, and while the diet contained moderate levels of linoleic acid, the arachidonic acid content was low (<0.5%). As such, the reduction in linoleic acid over time may be evidence of its use to synthesize arachidonic acid by way of delta 6 desaturase, elongase, and delta 5 desaturase (40). However, with % composition of arachidonic acid also decreasing over time and as the dogs trained and specifically in the CE and PL fractions, the demand for downstream metabolites of arachidonic acid, such as eicosanoids, appeared to have been greater than the supply from linoleic acid and/or the diet. When released from membrane PL, arachidonic acid can be converted to an array of different eicosanoids, such as prostaglandins, many of which are involved in the promotion of inflammatory responses, including exercise-induced inflammatory responses (41). Considering that the dogs in the present study were exercising 4 days a week for 12 weeks, it is likely that they were experiencing some degree of acute and/or chronic inflammation. These data may indicate that as exercise conditioning continues, that endurance training dogs may benefit from supplemental omega 6 FA; however, the changes observed in FA profiles over time in this study may alternatively suggest that a diet acclimation period of >4 weeks [identified with Alaskan huskies by (16); mixed breed dogs by (31), and cats by (32)] may be necessary to capture diet-induced changes to lipid profiles for adult Siberian huskies. Angle et al. (42) investigated the effects of exercise and diet (high fat vs. low fat vs. high PUFA) on olfactory capability in certified scent detection dogs and fed each diet for 8 weeks so as to allow for “metabolic adjustments to the diet” prior to olfaction testing. While differences in olfactory acuity were observed, with dogs on high PUFA diet being 1.4 times as likely to find a target than those on the high fat diet, there was never any support or evidence provided as to why this 8-week adaptation period was necessary (42). However, if a dietary adaptation of 8 weeks is necessary for exercising or performance dogs when investigating outcomes related to lipid metabolism, perhaps this may lend some support as to why changes over time, particularly for the essential PUFA linoleic acid (18:2n6) were more evident at 11 weeks rather than at 5 weeks.

A limitation of this study that should be acknowledged is that since these dogs experienced changes in body composition (increase in lean mass, decrease in fat mass, but no change in overall BW; 27) throughout the study period, it is not possible to differentiate between the effect of diet and the effect of exercise-induced changes to endogenous fat stores. Unfortunately, there is little information available on the diet (Redpaw PowerEdge 32k, Redpaw Inc., Franklin, WI) the dogs were fed prior to this study. All that is known is the minimal amount of crude fat (20%) from the guaranteed analysis, but the authors did not have access to data on the true (analyzed) fat content and/or FA composition of the diet. As such, no inferences can be drawn regarding how or if the FA composition of this diet contributed to changes observed over time in the present study. An additional limitation was the lack of a control group of dogs that remained sedentary throughout the study period. Having the ability to compare FA profiles between sedentary and actively-training dogs with all other management conditions being maintained would have provided insight into how or if the changes in FA profiles throughout the study period were a result of diet, exercise, environment, or a combination of these factors. However, when working with a cohort of client owned sled dogs preparing for a racing season, this was simply not feasible.

There is value in assessing FA composition of individual lipid fractions as these pools differ considerably in FA composition and each play distinct physiological roles (30). In fact, diversity of lipid fractions can be used to indicate the development or attenuation of various metabolic conditions, such as diabetes and obesity, in human and canine subjects alike (43–46); although, these outcomes were not necessarily within scope for this project as all dogs participating were healthy. With regard to assessing FA composition in healthy, exercising dogs, serum FFA has been examined most commonly (12, 21, 35), as it is known to be the most labile fraction during exercise. While the data presented herein will help to partially fill that void, future research evaluating changes in FA composition within lipid fractions in response to extended bouts of submaximal exercise is warranted. Furthermore, it would be of interest to assess these changes in FA composition within distinct lipid fractions of other tissues, such as adipose or muscle, alongside of serum, as these data may bridge our understanding of lipid mobilization and muscle metabolism in the exercising dog. Finally, the authors acknowledge that by only determining and reporting FA composition of individual lipid fractions as % of total peak area, that changes in FA composition should be interpreted with caution. While these data are valuable considering the dearth of available literature in this area, particularly with endurance trained sled dogs, it should be recognized that when expressing the values as % composition, each analyzed FA has the potential to influence the relative % of any/all other FA, as opposed to expressing the values as concentrations, where changes in FA are independent of each other (30, 47). As well, it should be recognized that an assessment of FA composition in serum simply provides an indication as to what is occurring in blood at that moment of time, essentially representing the balance between FA release into circulation and/or uptake into tissue. These samples do not represent kinetic or dynamic measurements of FA mobilization, and as such, it is important to keep this limitation in mind when interpreting changes (or lack thereof) in FA composition.

## Conclusions

The results from this study indicate that aside from reductions in % composition of oleic, linoleic, and alpha linolenic acids observed in the FFA lipid fraction, short bouts of submaximal exercise do not induce considerable changes to sled dog FA profiles. Moreover, depletion of these select 18-carbon unsaturated FA are comparable to changes observed in less endurance-specialized breeds subjected to exercise of similar intensity and duration. Changes in FA profiles in all lipid fractions aside from DAG were observed throughout the duration of the study; however, due to a lack of a sedentary control group of dogs, it is not possible to determine if these outcomes were a result of diet (e.g., insufficient dietary adaptation) or exercise training (e.g., reductions in endogenous lipid stores). While the data presented herein helps to improve our understanding of lipid utilization in sled dogs, future research investigating changes in circulating FA as well as gluconeogenic precursors (e.g., amino acids, lactate, glycerol) in both exercising and sedentary sled dogs is warranted to elucidate the extent to which these endurance-trained dogs rely on lipid oxidation during exercise.

## Data Availability Statement

The raw data supporting the conclusions of this article will be made available by the authors, without undue reservation.

## Ethics Statement

The animal study was reviewed and approved by University of Guelph's Animal Care Committee (animal use protocol #4008). Written informed consent was obtained from the owners for the participation of their animals in this study.

## Author Contributions

JRT and AKS designed research. JRT conducted research. JRT, LT, DWLM, and AKS analyzed the data and wrote the manuscript. AKS had primary responsibility for the final content. All authors have read and approved the final manuscript.

## Conflict of Interest

The authors declare that the research was funded by Champion Petfoods LT. (Morinville, AB) and Mitacs (Toronto, ON), which could be construed as a potential conflict of interest; however, the authors did not receive any personal financial incentives.

## Publisher's Note

All claims expressed in this article are solely those of the authors and do not necessarily represent those of their affiliated organizations, or those of the publisher, the editors and the reviewers. Any product that may be evaluated in this article, or claim that may be made by its manufacturer, is not guaranteed or endorsed by the publisher.
