# Supplementary material for: Fatty Acid Profiles of Serum Lipid Fractions Change Minimally in Sled Dogs Before and After Short Bouts of Exercise
Source: Front Vet Sci. 2021 Aug 23;8:704770. doi: 10.3389/fvets.2021.704770 (PMC8421022; doi:10.3389/fvets.2021.704770)
Supplement: Supplementary file 1 [file Data_Sheet_1.PDF]

Supplementary Table 1. Mean fatty acid composition (± SE[1]) for the cholesterol ester (CE) fraction identified in serum of all dogs at the pre- and post-exercise sampling time points for weeks 2, 5, and 11.

Fatty acid[2], %	Week 2		Week 5		Week 11	
	Pre	Post	Pre	Post	Pre	Post
12:0	2.52 ± 0.54	1.83 ± 0.54	1.65 ± 0.54	0.97 ± 0.54	1.73 ± 0.61	0.54 ± 0.61
14:0	0.11 ± 0.05	0.07 ± 0.05	0.06 ± 0.05	0.04 ± 0.05	0.07 ± 0.06	0.05 ± 0.06
15:0	0.05 ± 0.03	0.03 ± 0.03	0.03 ± 0.03	0.02 ± 0.03	0.04 ± 0.04	0.01 ± 0.04
16:0	10.72 ± 0.44	10.41 ± 0.44	11.13 ± 0.44	11.16 ± 0.44	15.15 ± 0.53	14.93 ± 0.53
18:0	4.75 ± 1.31	3.99 ± 1.31	5.51 ± 1.31	5.44 ± 1.31	5.69 ± 1.44	5.88 ± 1.44
19:0	0.09 ± 0.03	0.08 ± 0.03	0.03 ± 0.03	0.04 ± 0.03	0.03 ± 0.04	0.04 ± 0.04
22:0	0.21 ± 0.16	0.13 ± 0.16	0.21 ± 0.16	0.17 ± 0.16	0.40 ± 0.21	0.31 ± 0.21
23:0	0.09 ± 0.09	0.20 ± 0.09	0.10 ± 0.09	0.07 ± 0.09	0.04 ± 0.11	0.02 ± 0.11
24:0	0.23 ± 0.17	0.21 ± 0.17	0.19 ± 0.17	0.28 ± 0.17	0.26 ± 0.20	0.28 ± 0.20
14:1	0.46 ± 0.10	0.35 ± 0.10	0.17 ± 0.10	0.09 ± 0.10	0.05 ± 0.13	0.04 ± 0.13
16:1n7	1.26 ± 0.29	1.64 ± 0.29	2.04 ± 0.29	2.47 ± 0.29	2.65 ± 0.34	2.83 ± 0.34
17:1n7	0.03 ± 0.03	0.06 ± 0.03	0.04 ± 0.03	0.03 ± 0.03	0.03 ± 0.03	0.05 ± 0.03
18:1n7	3.42 ± 0.16	3.31 ± 0.16	3.12 ± 0.16	2.96 ± 0.16	2.74 ± 0.21	2.68 ± 0.21
18:1n9	14.32 ± 0.55	13.69 ± 0.55	12.45 ± 0.55	12.91 ± 0.55	13.57 ± 0.64	14.02 ± 0.64
20:1n11	0.12 ± 0.06	0.08 ± 0.06	0.10 ± 0.06	0.09 ± 0.06	0.13 ± 0.09	0.11 ± 0.09
22:1n9	1.12 ± 0.31	1.02 ± 0.31	0.98 ± 0.31	0.87 ± 0.31	1.29 ± 0.38	1.01 ± 0.38
24:1	0.72 ± 0.15	0.93 ± 0.15	0.71 ± 0.15	0.64 ± 0.15	0.61 ± 0.15	0.59 ± 0.15
18:2n6	50.15 ± 2.13	51.60 ± 2.13	48.72 ± 2.13	50.28 ± 2.13	41.72 ± 2.46	43.05 ± 2.46
18:3n6	0.05 ± 0.08	0.21 ± 0.08	0.23 ± 0.08	0.09 ± 0.08	0.09 ± 0.11	0.05 ± 0.11
20:2n6	0.03 ± 0.05	0.05 ± 0.05	0.05 ± 0.05	0.05 ± 0.05	0.04 ± 0.07	0.14 ± 0.07
20:4n6	14.41 ± 0.71	14.70 ± 0.71	11.71 ± 0.71	11.81 ± 0.71	9.48 ± 0.88	10.22 ± 0.88
22:4n6	0.02 ± 0.02	0.03 ± 0.02	0.03 ± 0.02	0.06 ± 0.02	0.07 ± 0.03	0.20 ± 0.03
18:3n3	0.06 ± 0.08	0.02 ± 0.08	0.17 ± 0.08	0.12 ± 0.08	0.22 ± 0.11	0.15 ± 0.11
20:5n3	1.59 ± 0.28	1.44 ± 0.28	1.67 ± 0.28	1.41 ± 0.28	1.86 ± 0.36	1.74 ± 0.36
22:6n3	0.39 ± 0.24	0.35 ± 0.24	0.43 ± 0.24	0.39 ± 0.24	0.52 ± 0.29	0.48 ± 0.29

[1]SE, standard error of the mean; n = 15 for weeks 2 and 5; n = 13 for week 11.
[2]Any fatty acid(s) not detected within the specific fraction were removed from the table.
†Mean value for the pre-exercise sampling time point significantly differs from the post-exercise sampling time point within the same week ($P \leq 0.05$).

Supplementary Table 2. Mean fatty acid composition (± SE[1]) for the diacylglycerol (DAG) fraction identified in serum of all dogs at the pre- and post-exercise sampling time points for weeks 2, 5, and 11.

Fatty acid[2], %	Week 2		Week 5		Week 11	
	Pre	Post	Pre	Post	Pre	Post
14:0	0.04 ± 0.04	0.02 ± 0.04	0.05 ± 0.04	0.01 ± 0.04	0.05 ± 0.05	0.01 ± 0.05
16:0	28.82 ± 3.89	27.44 ± 3.89	32.79 ± 3.89	31.19 ± 3.89	32.58 ± 4.40	30.96 ± 4.40
18:0	22.56 ± 3.12	26.67 ± 3.12	21.41 ± 3.12	23.11 ± 3.12	21.58 ± 3.46	22.43 ± 3.46
19:0	0.18 ± 0.15	0.26 ± 0.15	0.02 ± 0.15	0.06 ± 0.15	0.06 ± 0.18	0.08 ± 0.18
22:0	0.22 ± 0.15	0.18 ± 0.15	0.31 ± 0.15	0.23 ± 0.15	0.26 ± 0.17	0.19 ± 0.17
23:0	0.28 ± 0.18	0.17 ± 0.18	0.14 ± 0.18	0.12 ± 0.18	0.16 ± 0.21	0.09 ± 0.21
16:1n7	1.34 ± 1.15	1.18 ± 1.15	1.92 ± 1.15	1.63 ± 1.15	1.86 ± 1.29	1.67 ± 1.29
18:1n9	15.84 ± 2.84	20.38 ± 2.84	19.62 ± 2.84	22.27 ± 2.84	19.59 ± 3.01	22.08 ± 3.01
18:1n7	1.18 ± 1.17	2.13 ± 1.17	0.65 ± 1.17	1.44 ± 1.17	0.50 ± 1.29	1.49 ± 1.29
20:1n11	0.04 ± 0.05	0.02 ± 0.05	0.05 ± 0.05	0.02 ± 0.05	0.05 ± 0.06	0.02 ± 0.06
22:1n9	1.42 ± 0.92	1.01 ± 0.92	1.53 ± 0.92	1.17 ± 0.92	1.48 ± 0.99	1.20 ± 0.99
24:1	1.12 ± 0.70	0.97 ± 0.70	0.81 ± 0.70	0.65 ± 0.70	0.82 ± 0.70	0.48 ± 0.70
18:2n6	5.66 ± 1.41	5.20 ± 1.41	5.58 ± 1.41	5.38 ± 1.41	7.76 ± 1.56	7.39 ± 1.56
18:3n6	0.12 ± 0.13	0.30 ± 0.13	0.11 ± 0.13	0.24 ± 0.13	0.03 ± 0.15	0.09 ± 0.15
20:4n6	2.94 ± 0.89	2.31 ± 0.89	2.16 ± 0.89	1.40 ± 0.89	2.50 ± 0.96	1.92 ± 0.96
18:3n3	0.02 ± 0.03	0.01 ± 0.03	0.02 ± 0.03	0.01 ± 0.03	0.03 ± 0.04	0.01 ± 0.04
20:5n3	0.51 ± 0.24	0.34 ± 0.24	0.39 ± 0.24	0.29 ± 0.24	0.36 ± 0.30	0.34 ± 0.30
22:6n3	0.50 ± 0.25	0.30 ± 0.25	0.31 ± 0.25	0.18 ± 0.25	0.34 ± 0.30	0.23 ± 0.30

[1]SE, standard error of the mean; n = 15 for weeks 2 and 5; n = 13 for week 11.
[2]Any fatty acid(s) not detected within the specific fraction were removed from the table.
[†]Mean value for the pre-exercise sampling time point significantly differs from the post-exercise sampling time point within the same week ($P \leq 0.05$).

Supplementary Table 3. Mean fatty acid composition (± SE[1]) for the free fatty acid (FFA) fraction identified in serum of all dogs at the pre- and post-exercise sampling time points for weeks 2, 5, and 11.

Fatty acid[2], %	Week 2		Week 5		Week 11	
	Pre	Post	Pre	Post	Pre	Post
14:0	0.13 ± 0.09	0.27 ± 0.09	0.06 ± 0.09	0.14 ± 0.09	0.07 ± 0.12	0.12 ± 0.12
15:0	0.01 ± 0.01	0.01 ± 0.01	0.02 ± 0.01	0.02 ± 0.01	0.01 ± 0.02	0.01 ± 0.02
16:0	25.36 ± 1.39	21.84 ± 1.39	25.35 ± 1.39	24.70 ± 1.39	32.04 ± 1.81	28.03 ± 1.81
18:0	11.12 ± 0.93	9.50 ± 0.93	11.86 ± 0.93	11.05 ± 0.93	15.46 ± 1.21	14.33 ± 1.21
24:0	0.03 ± 0.07	0.07 ± 0.07	0.02 ± 0.07	0.08 ± 0.07	0.03 ± 0.10	0.06 ± 0.10
14:1	0.18 ± 0.12	0.22 ± 0.12	0.14 ± 0.12	0.22 ± 0.12	0.04 ± 0.17	0.06 ± 0.17
16:1n7	3.14 ± 0.57	4.02 ± 0.57	4.21 ± 0.57	5.07 ± 0.57	5.63 ± 0.81	5.74 ± 0.81
18:1n9	33.42 ± 1.78	31.47 ± 1.78	31.72 ± 1.78	26.90 ± 1.78	32.85 ± 2.20	29.53 ± 2.20
18:1n7	3.49 ± 0.29	3.62 ± 0.29	2.60 ± 0.29	2.69 ± 0.29	2.31 ± 0.41	2.82 ± 0.41
20:1n11	0.03 ± 0.05	0.05 ± 0.05	0.01 ± 0.05	0.04 ± 0.05	0.03 ± 0.08	0.05 ± 0.08
22:1n9	0.92 ± 0.46	0.60 ± 0.46	1.01 ± 0.46	0.76 ± 0.46	1.23 ± 0.72	0.97 ± 0.72
24:1	0.14 ± 0.12	0.06 ± 0.12	0.11 ± 0.12	0.02 ± 0.12	0.09 ± 0.18	0.05 ± 0.18
18:2n6	18.63 ± 0.99	16.59 ± 0.99	13.64 ± 0.99	12.71 ± 0.99	12.91 ± 1.34	11.38 ± 1.34
20:4n6	1.54 ± 0.41	1.19 ± 0.41	1.38 ± 0.41	1.06 ± 0.41	1.44 ± 0.62	1.12 ± 0.62
22:5n6	2.72 ± 1.44	2.02 ± 1.44	3.19 ± 1.44	2.00 ± 1.44	5.21 ± 1.82	3.46 ± 1.82
18:3n3	0.89 ± 0.13	0.44 ± 0.13	0.70 ± 0.13	0.26 ± 0.13	0.35 ± 0.16	0.08 ± 0.16
20:5n3	0.04 ± 0.07	0.08 ± 0.07	0.06 ± 0.07	0.08 ± 0.07	0.02 ± 0.09	0.06 ± 0.09
22:6n3	0.26 ± 0.16	0.41 ± 0.16	0.27 ± 0.16	0.37 ± 0.16	0.12 ± 0.31	0.18 ± 0.31

[1]SE, standard error of the mean; n = 15 for weeks 2 and 5; n = 13 for week 11.
[2]Any fatty acid(s) not detected within the specific fraction were removed from the table.
[†]Mean value for the pre-exercise sampling time point significantly differs from the post-exercise sampling time point within the same week ($P \leq 0.05$).

Supplementary Table 4. Mean fatty acid composition (± SE[1]) for the phospholipid (PL) fraction identified in serum of all dogs at the pre- and post-exercise sampling time points for weeks 2, 5, and 11.

Fatty acid[2], %	Week 2		Week 5		Week 11	
	Pre	Post	Pre	Post	Pre	Post
14:0	0.10 ± 0.03	0.08 ± 0.03	0.09 ± 0.03	0.07 ± 0.03	0.09 ± 0.04	0.07 ± 0.04
15:0	0.07 ± 0.02	0.06 ± 0.02	0.07 ± 0.02	0.07 ± 0.02	0.03 ± 0.02	0.04 ± 0.02
16:0	15.23 ± 0.49	16.10 ± 0.49	17.12 ± 0.49	17.32 ± 0.49	15.95 ± 0.58	16.87 ± 0.58
18:0	25.07 ± 2.01	23.00 ± 2.01	22.22 ± 2.01	19.72 ± 2.01	16.32 ± 2.26	14.73 ± 2.26
19:0	0.17 ± 0.05	0.13 ± 0.05	0.13 ± 0.05	0.12 ± 0.05	0.08 ± 0.07	0.07 ± 0.07
20:0	0.10 ± 0.04	0.10 ± 0.04	0.09 ± 0.04	0.13 ± 0.04	0.15 ± 0.06	0.18 ± 0.06
22:0	0.06 ± 0.06	0.10 ± 0.06	0.09 ± 0.06	0.12 ± 0.06	0.14 ± 0.08	0.20 ± 0.08
23:0	0.12 ± 0.06	0.11 ± 0.06	0.12 ± 0.06	0.12 ± 0.06	0.05 ± 0.07	0.06 ± 0.07
24:0	0.11 ± 0.05	0.13 ± 0.05	0.11 ± 0.05	0.10 ± 0.05	0.12 ± 0.07	0.16 ± 0.07
14:1	0.01 ± 0.02	0.01 ± 0.02	0.02 ± 0.02	0.02 ± 0.02	0.02 ± 0.03	0.02 ± 0.03
16:1n7	0.56 ± 0.23	0.50 ± 0.23	0.69 ± 0.23	0.62 ± 0.23	1.37 ± 0.38	1.16 ± 0.38
17:1n7	0.25 ± 0.05	0.23 ± 0.05	0.29 ± 0.05	0.29 ± 0.05	0.25 ± 0.07	0.17 ± 0.07
18:1n7	2.98 ± 0.41	3.11 ± 0.41	2.34 ± 0.41	2.75 ± 0.41	2.41 ± 0.55	2.66 ± 0.55
18:1n9	8.59 ± 0.94	9.02 ± 0.94	8.57 ± 0.94	9.96 ± 0.94	10.27 ± 1.23	10.78 ± 1.23
20:1n11	0.22 ± 0.05	0.21 ± 0.05	0.22 ± 0.05	0.18 ± 0.05	0.19 ± 0.06	0.18 ± 0.06
22:1n9	0.44 ± 0.19	0.51 ± 0.19	0.47 ± 0.19	0.50 ± 0.19	0.51 ± 0.24	0.72 ± 0.24
24:1	0.97 ± 0.09	1.05 ± 0.09	0.79 ± 0.09	0.77 ± 0.09	0.54 ± 0.13	0.68 ± 0.13
20:3n9	0.11 ± 0.04	0.12 ± 0.04	0.09 ± 0.04	0.10 ± 0.04	0.05 ± 0.06	0.08 ± 0.06
18:2n6	26.54 ± 1.73	26.12 ± 1.73	24.62 ± 1.73	24.27 ± 1.73	20.22 ± 2.06	19.98 ± 2.06
18:3n6	0.18 ± 0.05	0.13 ± 0.05	0.18 ± 0.05	0.17 ± 0.05	0.12 ± 0.07	0.08 ± 0.07
20:2n6	0.10 ± 0.04	0.16 ± 0.04	0.14 ± 0.04	0.18 ± 0.04	0.22 ± 0.05	0.24 ± 0.05
20:3n6	0.83 ± 0.11	1.21 ± 0.11	0.88 ± 0.11	1.19 ± 0.11	0.63 ± 0.14	0.81 ± 0.14
20:4n6	17.05 ± 0.82	17.21 ± 0.82	15.38 ± 0.82	16.12 ± 0.82	14.94 ± 1.03	15.49 ± 1.03
22:4n6	0.21 ± 0.05	0.20 ± 0.05	0.20 ± 0.05	0.23 ± 0.05	0.26 ± 0.07	0.29 ± 0.07
22:5n6	0.11 ± 0.02	0.12 ± 0.02	0.09 ± 0.02	0.11 ± 0.02	0.10 ± 0.03	0.10 ± 0.03
18:3n3	0.33 ± 0.05	0.28 ± 0.05	0.35 ± 0.05	0.26 ± 0.05	0.18 ± 0.06	0.16 ± 0.06
20:5n3	2.40 ± 0.26	2.31 ± 0.26	2.70 ± 0.26	2.63 ± 0.26	2.44 ± 0.37	2.56 ± 0.37
22:5n3	0.57 ± 0.09	0.64 ± 0.09	0.52 ± 0.09	0.58 ± 0.09	0.72 ± 0.16	0.77 ± 0.16
22:6n3	3.46 ± 0.41	3.54 ± 0.41	2.34 ± 0.41	2.37 ± 0.41	3.20 ± 0.55	3.45 ± 0.55

[1]SE, standard error of the mean; n = 15 for weeks 2 and 5; n = 13 for week 11.

[2]Any fatty acid(s) not detected within the specific fraction were removed from the table.

[†]Mean value for the pre-exercise sampling time point significantly differs from the post-exercise sampling time point within the same week ($P \leq 0.05$).

Supplementary Table 5. Mean fatty acid composition (± SE[1]) for the triacylglycerol (TAG) fraction identified in serum of all dogs at the pre- and post-exercise sampling time points for weeks 2, 5, and 11.

Fatty acid[2], %	Week 2		Week 5		Week 11	
	Pre	Post	Pre	Post	Pre	Post
14:0	0.35 ± 0.13	0.50 ± 0.13	0.27 ± 0.13	0.34 ± 0.13	0.19 ± 0.15	0.22 ± 0.15
15:0	0.01 ± 0.03	0.01 ± 0.03	0.01 ± 0.03	0.02 ± 0.03	0.01 ± 0.04	0.01 ± 0.04
16:0	22.01 ± 1.44	19.40 ± 1.44	21.90 ± 1.44	21.13 ± 1.44	30.11 ± 1.77	31.41 ± 1.77
18:0	10.93 ± 1.26	11.24 ± 1.26	9.03 ± 1.26	10.07 ± 1.26	21.50 ± 1.41	21.81 ± 1.41
20:0	0.15 ± 0.13	0.12 ± 0.13	0.11 ± 0.13	0.12 ± 0.13	0.31 ± 0.15	0.20 ± 0.15
22:0	0.05 ±0.06	0.06 ± 0.06	0.04 ± 0.06	0.05 ± 0.06	0.13 ± 0.07	0.07 ± 0.07
24:0	0.12 ± 0.12	0.20 ± 0.12	0.14 ± 0.12	0.22 ± 0.12	0.06 ± 0.16	0.08 ± 0.16
14:1	0.10 ± 0.15	0.10 ± 0.15	0.25 ± 0.15	0.12 ± 0.15	0.33 ± 0.26	0.24 ± 0.26
16:1n7	2.61 ± 0.71	2.18 ± 0.71	3.87 ± 0.71	3.39 ± 0.71	4.39 ± 0.84	3.93 ± 0.84
18:1n7	2.81 ± 0.42	4.29 ± 0.42	3.69 ± 0.42	3.81 ± 0.42	2.22 ± 0.55	2.75 ± 0.55
18:1n9	31.81 ± 1.77	29.49 ± 1.77	33.21 ± 1.77	33.55 ± 1.77	18.49 ± 2.03	20.96 ± 2.03
20:1n11	0.06 ± 0.05	0.03 ± 0.05	0.05 ± 0.05	0.03 ± 0.05	0.08 ± 0.06	0.02 ± 0.06
22:1n9	1.68 ± 1.33	1.87 ± 1.33	3.55 ± 1.33	3.79 ± 1.33	3.13 ± 1.49	3.37 ± 1.49
24:1	0.20 ± 0.10	0.13 ± 0.10	0.11 ± 0.10	0.05 ± 0.10	0.13 ± 0.13	0.01 ± 0.13
18:2n6	18.49 ± 1.15	18.31 ± 1.15	15.01 ± 1.15	14.93 ± 1.15	10.04 ± 1.33	9.98 ± 1.33
20:3n6	0.18 ± 0.07	0.12 ± 0.07	0.14 ± 0.07	0.09 ± 0.07	0.16 ± 0.09	0.08 ± 0.09
20:4n6	4.81 ± 0.72	5.16 ± 0.72	3.22 ± 0.72	3.53 ± 0.72	3.34 ± 0.89	4.07 ± 0.89
22:4n6	0.01 ± 0.01	0.01 ± 0.01	0.02 ± 0.01	0.01 ± 0.01	0.01 ± 0.02	0.01 ± 0.02
18:3n3	0.33 ± 0.11	0.27 ± 0.11	0.23 ± 0.11	0.24 ± 0.11	0.27 ± 0.16	0.22 ± 0.16
20:5n3	1.91 ± 0.52	2.01 ± 0.52	1.59 ± 0.52	1.77 ± 0.52	1.48 ± 0.62	2.17 ± 0.62
22:5n3	0.09 ± 0.08	0.05 ± 0.08	0.08 ± 0.08	0.06 ± 0.08	0.07 ± 0.11	0.03 ± 0.11
22:6n3	2.41 ± 0.63	2.18 ± 0.63	1.95 ± 0.63	2.46 ± 0.63	1.80 ± 0.77	2.08 ± 0.77

[1]SE, standard error of the mean; n = 15 for weeks 2 and 5; n = 13 for week 11.
[2]Any fatty acid(s) not detected within the specific fraction were removed from the table.
[†]Mean value for the pre-exercise sampling time point significantly differs from the post-exercise sampling time point within the same week ($P \leq 0.05$).
